# RbdB, a Rhomboid Protease Critical for SREBP Activation and Virulence in *Aspergillus fumigatus*

**DOI:** 10.1128/mSphere.00035-16

**Published:** 2016-03-02

**Authors:** Sourabh Dhingra, Caitlin H. Kowlaski, Arsa Thammahong, Sarah R. Beattie, Katherine M. Bultman, Robert A. Cramer

**Affiliations:** Department of Microbiology and Immunology, Geisel School of Medicine at Dartmouth, Hanover, New Hampshire, USA; Yonsei University

**Keywords:** *Aspergillus fumigatus*, antifungal agents, fungal pathogenesis, hypoxia, fungal virulence, sterol regulatory element binding protein

## Abstract

SREBP transcription factors play a critical role in fungal virulence; however, the mechanisms of sterol regulatory element binding protein (SREBP) activation in pathogenic fungi remains ill-defined. Screening of the *Neurospora crassa* whole-genome deletion collection for genes involved in hypoxia responses identified a gene for an uncharacterized rhomboid protease homolog, *rbdB*, required for growth under hypoxic conditions. Loss of *rbdB* in *Aspergillus fumigatus* also inhibited growth under hypoxic conditions. In addition, the *A. fumigatus* Δ*rbdB* strain also displayed phenotypes consistent with defective SREBP activity, including increased azole drug susceptibility, reduced siderophore production, and full loss of virulence. Expression of the basic helix-loop-helix (bHLH) DNA binding domain of the SREBP SrbA in Δ*rbdB* restored all of the phenotypes linking RdbB activity with SrbA function. Furthermore, the N-terminal domain of SrbA containing the bHLH DNA binding region was absent from Δ*rbdB* under inducing conditions, suggesting that RbdB regulates the protein levels of this important transcription factor. As SrbA controls clinically relevant aspects of fungal pathobiology in *A. fumigatus*, understanding the mechanisms of SrbA activation provides opportunities to target this pathway for therapeutic development.

**IMPORTANCE** *Aspergillus fumigatus* causes life-threatening infections, and treatment options remain limited. Thus, there is an urgent need to find new therapeutic targets to treat this deadly disease. Previously, we have shown that SREBP transcription factors and their regulatory components are critical for the pathobiology of *A. fumigatus*. Here we identify a role for RbdB, a rhomboid protease, as an essential component of SREBP activity. Our results indicate that mutants lacking *rbdB* have growth defects under hypoxic conditions, are hypersusceptible to voriconazole, lack extracellular siderophore production, and fail to cause disease in a murine model of invasive pulmonary aspergillosis. This study increases our understanding of the molecular mechanisms involved in SREBP activation in pathogenic fungi and provides a novel therapeutic target for future development.

## Abstract (precis)

*Aspergillus fumigatus* causes life-threatening infections, and treatment options remain limited. Thus, there is an urgent need to find new therapeutic targets to treat this deadly disease. Previously, we have shown that SREBP transcription factors and their regulatory components are critical for the pathobiology of *A. fumigatus*. Here we identify a role for RbdB, a rhomboid protease, as an essential component of SREBP activity. Our results indicate that mutants lacking *rbdB* have growth defects under hypoxic conditions, are hypersusceptible to voriconazole, lack extracellular siderophore production, and fail to cause disease in a murine model of invasive pulmonary aspergillosis. This study increases our understanding of the molecular mechanisms involved in SREBP activation in pathogenic fungi and provides a novel therapeutic target for future development.

## INTRODUCTION

Fungal sterol regulatory element binding protein (SREBP) transcription factors in the human-pathogenic fungi *Cryptococcus neoformans* and *Aspergillus fumigatus* are essential for virulence (1 – 3). The *A. fumigatus* SREBP SrbA is critical for adaptation and growth under hypoxic conditions, responses to triazole antifungal drug stress, and optimal iron acquisition (1, 4). Through its genetic interaction with another SREBP family member, SrbB, SrbA also mediates key central metabolism pathways impacted by oxygen availability, such as ethanol fermentation, oxidative phosphorylation, and heme biosynthesis (5).

Regulation of SREBP activity in fungi has been extensively studied in the fission yeast *Schizosaccharomyces pombe* (6 – 9). Seminal studies with *S. pombe* revealed that the SREBP pathway functions as an indirect oxygen sensor through its monitoring of ergosterol levels in the cell (6). Key to this molecular mechanism is the presence of the sterol cleavage-activating protein, SCAP, which senses ergosterol levels (6). When sterol levels are replete, SCAP binds to the yeast SREBP Sre1, maintaining Sre1 in the endoplasmic reticulum (ER) and preventing regulated intramembrane proteolysis (RIP). RIP of SREBPs in mammals releases the N-terminal domain of SREBPs that function as transcription factors to regulate cholesterol and lipid homeostasis (10).

In *S. pombe*, SCAP null mutants phenocopy *sre1* null mutants in their inability to grow under hypoxic conditions (6). A similar phenotype is observed in the human-pathogenic fungus *C. neoformans* (2, 3). In *C. neoformans*, a site 2 protease was identified that releases the N-terminal portion of Sre1 in response to sterol depletion (11). In *S. pombe* and *A. fumigatus*, the identity of the protease regulating Sre1/SrbA transcription factor activity has remained elusive. A genetic screen of *S. pombe* identified members of a novel Golgi Dsc E3 ligase complex required for Sre1 processing (7). Despite the absence of SCAP in *A. fumigatus*, Dsc ortholog function in *A. fumigatus* is conserved and linked to regulation of SREBP activity in this pathogenic mold (12). Recently, another genetic screen of the *S. pombe* genome deletion collection identified a novel rhomboid family protease critical for regulation of Sre1 function in this yeast, Rbd2 (13).

Here we report that screening of the *Neurospora crassa* whole-genome deletion collection identified an *rbd2* ortholog essential for growth under hypoxic conditions in this model filamentous fungus. We then generated an *rbd2* (here *rbdB*) genetic null mutant of *A. fumigatus* and observed that RbdB is essential for SrbA activity in *A. fumigatus*. The *A. fumigatus rbdB* null mutant phenocopies the *srbA* null mutant with regard to loss of growth under hypoxic conditions, enhanced antifungal drug susceptibility, decreased siderophore production, and full loss of virulence. Discovery of a protease critical for SrbA function presents a new opportunity to further define the molecular mechanism of SrbA activity in this important pathogenic fungus and uncover potential novel fungus-specific therapeutic opportunities.

## RESULTS

### *rbd2* is necessary for *N. crassa* adaptation to hypoxia.

**FIG 1:**
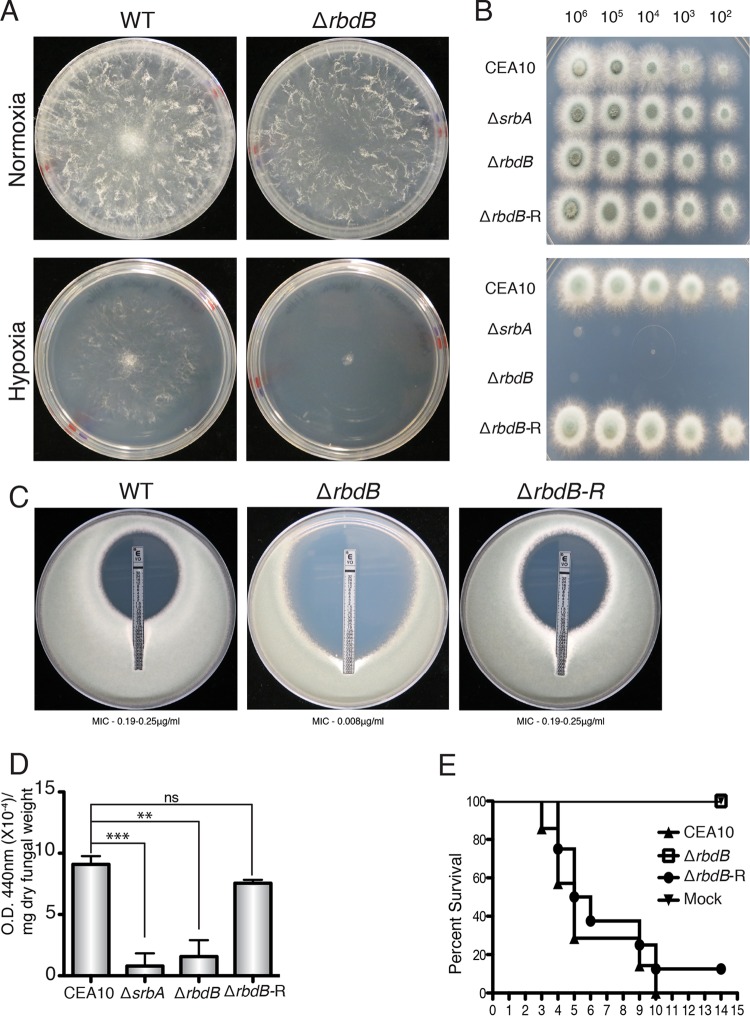
RbdB is critical for *A. fumigatus* growth under hypoxic conditions, voriconazole susceptibility, iron homeostasis, and virulence. (A) Growth of WT *N. crassa* and the Δ*rbd2* strain (10^3^ spores/plate) on Vogel’s minimal medium under normoxic and hypoxic conditions (0.2% O_2_, 5% CO_2_). Compared to the WT, Δ*rbd2* failed to grow under hypoxic conditions. (B) WT *A. fumigatus*, Δ*srbA*, Δ*rbdB*, and Δ*rbdB*-R strains were serially inoculated into GMM. Growth was observed after 48 h of incubation at 37°C in complete darkness. The WT and Δ*rbdB*-R strains showed similar growth under normoxic and hypoxic conditions, whereas Δ*srbA* and Δ*rbdB* did not demonstrate detectable growth under hypoxic conditions. (C) Voriconazole susceptibility was measured with Etest strips as described in Materials and Methods. The WT and Δ*rbdB*-R strains were susceptible to voriconazole (MIC, 0.19 to 0.25 µg/ml), whereas Δ*rbdB* showed increased susceptibility to voriconazole (MIC, 0.008 µg/ml). (D) Extracellular siderophore production was measured in the WT, Δ*srbA*, Δ*rbdB*, and Δ*rbdB*-R strains as described in Materials and Methods. Compared to the WT, Δ*srbA* showed a 90% reduction (*P* ≤ 0.001) and Δ*rbdB* showed an 80% reduction (*P* ≤ 0.01) in extracellular siderophore production. The Δ*rbdB*-R strain produced extracellular siderophore levels similar to those of the WT. (E) RbdB is indispensable for *A. fumigatus* virulence. Outbred CD-1 mice (*n* = 7 for the WT and Δ*rbdB* and *n* = 8 for the Δ*rbdB*-R strain) were immunosuppressed by subcutaneous injection of 40 mg/kg triamcinolone acetonide 1 day prior to inoculation. Mice were inoculated with 2 × 10^6^ spores of the WT, Δ*rbdB*, or Δ*rbdB*-R strains in 40 µl of PBS. Mouse survival was significantly different for Δ*rbdB* compared to the WT (*P* = 0.0001) and the Δ*rbdB*-R strain (*P* = 0.0009) by log rank tests. No significant difference was observed between the WT and the Δ*rbdB*-R strain (*P* = 0.3643). ***, *P* ≤ 0.001; **, *P* ≤ 0.01; ns, not significant. O.D., optical density.

As part of our screen of the *N. crassa* whole-genome deletion collection for genes involved in growth under hypoxic conditions, an *N. crassa* strain deficient in NCU02371.7 failed to germinate under hypoxic conditions (0.2% O_2_, 5% CO_2_) (Fig. 1A). Analysis of the amino acid sequence of NCU02371.7 revealed the presence of a peptidase S54 rhomboid domain. A recent study identified a similar rhomboid protease, Rbd2, in *S. pombe* as a candidate protease for yeast Sre1 cleavage (13). Sequence alignment of *N. crassa* NCU02371.7 and *S. pombe* Rbd2 suggested that NCU02371.7 is the *N. crassa* Rbd2 ortholog. A BLASTp search using the *N. crassa* Rbd2 amino acid sequence query against the *A. fumigatus* A1163 genome database revealed an uncharacterized gene, *AFUB_078750*, as a putative *rbd2* ortholog in this important human pathogen. Here, we refer to *AFUB_078750* as *rbdB*. On the basis of these observations, we sought to identify the role of RbdB in *A. fumigatus* adaptation to hypoxia and consequently generated genetic null mutant and reconstituted strains.

### *rbdB* is required for *A. fumigatus* adaptation to hypoxia.

To test the hypothesis that *rbdB* is required for *A. fumigatus* growth under hypoxic conditions, the wild-type (WT), Δ*rbdB*, and Δ*rbdB*-R strains were serially inoculated onto glucose minimal medium (GMM) (14) and allowed to grow for 48 h in the dark. After 48 h, Δ*rbdB* failed to germinate under hypoxic conditions, whereas the WT and Δ*rbdB*-R strains showed comparable growth under normoxic and hypoxic conditions (Fig. 1B). As Δ*rbdB* phenocopied the growth phenotype of Δ*srbA* under hypoxic conditions, we sought to determine the role of *rbdB* in other SrbA-dependent phenotypes.

### Loss of *rbdB* results in increased voriconazole susceptibility

We have previously shown that SrbA mediates triazole drug responses through direct binding of the *cyp51A* (*erg11A*) promoter region (1, 5, 15). Thus, we tested the voriconazole susceptibility of Δ*rbdB* by using voriconazole Etest strips. The WT and Δ*rbdB*-R strains showed susceptibility at voriconazole MICs previously reported for *A. fumigatus* (0.19 to 0.25 µg/ml). However, Δ*rbdB* showed a substantial increase in susceptibility to voriconazole (0.008 µg/ml) (Fig. 1C). This phenotype is similar to the previously reported susceptibility of Δ*srbA* to voriconazole (0.006 µg/ml) (1).

### *rbdB* is essential for iron homeostasis.

Iron is an essential cofactor for all living organisms, and its acquisition is essential for the growth and virulence of *A. fumigatus* (4, 16). *A. fumigatus* produces extracellular siderophores in response to iron starvation, and this production is, in part, SrbA dependent (4, 17). Thus, we sought to determine the effects of *rbdB* loss on extracellular siderophore production. Our results suggest an ~80% reduction in extracellular siderophore production in Δ*rbdB* compared to the WT (*P* ≤ 0.01), whereas the ability to produce extracellular siderophores was restored in the Δ*rbdB*-R strain (Fig. 1D). This reduction in extracellular siderophores was comparable to the levels of siderophores produced by Δ*srbA* (Fig. 1D). A potential limitation of this assay, however, is that both Δ*rbdB* and Δ*srbA* exhibit growth defects under low-iron and hypoxic conditions. While we normalized our data to the overall biomass of each strain, we cannot directly rule out indirect effects on iron homeostasis mechanisms in these respective null mutants in the interpretation of these data. Thus, similar to SrbA, RdbB is critical for growth under low-iron conditions and this effect may be mediated by perturbations in siderophore production in *A. fumigatus.*

### *rbdB* is indispensable for fungal virulence in a murine model of IPA.

Previous studies have indicated that SrbA is indispensable for virulence in *A. fumigatus* and *C. neoformans* (1, 2). As Δ*rbdB* phenocopied Δ*srbA* in virulence-associated *in vitro* stress screens, we next examined the virulence of Δ*rbdB* in a steroid murine model of invasive pulmonary aspergillosis (IPA) as previously described (18). Mice inoculated with WT CEA10 and the Δ*rbdB*-R strain started to die on day 4 after fungal challenges, and 100% and 87.5% mortality rates were observed, respectively, by day 10 (*P* = 0.3643). In contrast, mice challenged with Δ*rbdB* did not show any disease symptoms and survived the course of the experiment (*P* ≤ 0.0001 compared to the WT) (Fig. 1E). Thus, we conclude that *rbdB* is indispensable for fungal virulence. As Δ*rbdB* phenocopies Δ*srbA* with respect to growth under hypoxic conditions, voriconazole susceptibility, siderophore production, and virulence, we hypothesized that RdbB activity is upstream of SrbA and is essential for full SrbA activity in *A. fumigatus*.

### SrbA-dependent gene expression is positively regulated by *rbdB*.

**FIG 2:**
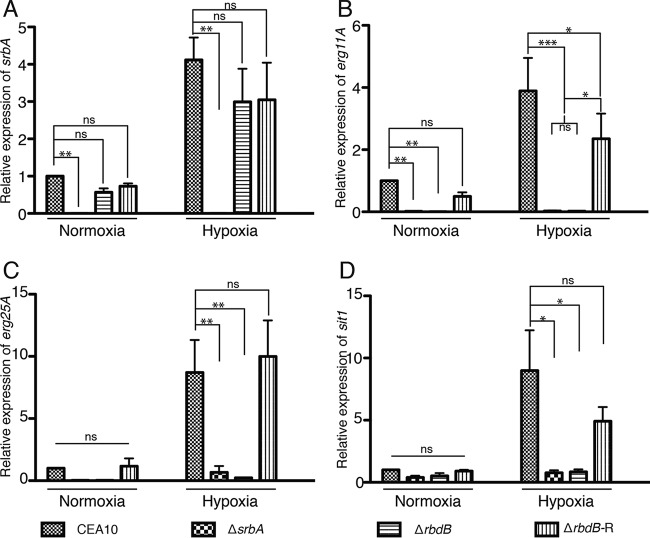
SrbA target gene mRNA levels are reduced in the absence RbdB. WT *A. fumigatus*, Δ*srbA*, Δ*rbdB*, and Δ*rbdB*-R strains were cultured in GMM at 37°C for 16 h; this was followed by a shift to hypoxia for 2 h. mRNA levels of SrbA target genes in the ergosterol biosynthetic pathway (*erg11A*, *erg25A*) and iron acquisition (*sit1*) were measured by quantitative RT-PCR. Data represent the mean and standard error of the mean of three biological replicates. *, *P* ≤ 0.05; **, *P* ≤ 0.01; ***, *P* ≤ 0.001; ns, nonsignificant (one-way ANOVA).

To test our hypothesis, we next examined the mRNA levels of transcripts known to be direct targets of SrbA activity. SrbA functions as a transcriptional activator and regulates a diverse array of clinically relevant functions in *A. fumigatus*, including adaptation to hypoxia, ergosterol biosynthesis, azole drug susceptibility, and iron acquisition (1, 4, 15, 18). To determine the role of RbdB on mRNA levels of known SrbA target genes, we compared the mRNA levels of *erg11A* (*cyp51A*), *erg25A* (ergosterol biosynthesis), and *sit1* (putative siderophore transporter) in the WT, Δ*rbdB*, and Δ*rbdB*-R strains by quantitative reverse transcription (RT)-PCR. As previously reported, we observed increased *erg11A*, *erg25A*, and *sit1* mRNA levels after shifting the WT and *rbdB*-R strain fungal cultures to hypoxia for 2 h. However, in Δ*rbdB*, the mRNA levels of these transcripts were significantly lower than those in the WT or the reconstituted strain under hypoxic conditions (Fig. 2B to D). We did observe a statistically significant difference in *erg11A* transcript levels between the WT and reconstituted strains, indicating that *erg11A* levels were not fully restored, perhaps because of the genomic location of the ectopically inserted allele. However, statistically significantly lower *erg11A* mRNA levels were observed in Δ*rbdB* than in the Δ*rbdB*-R strain. To determine if this reduction in mRNA levels of SrbA target transcripts is due to *srbA* transcript loss, we also tested the *srbA* mRNA levels in the WT, Δ*rbdB*, and Δ*rbdB*-R strains. Interestingly, *srbA* mRNA levels were comparable in all of the strains examined (Fig. 2A), suggesting that the effects of RdbB on SrbA are posttranscriptional. As posttranslational cleavage of SrbA is essential for SrbA-mediated functions in *A. fumigatus*, we next sought to determine the role of RbdB in SrbA protein levels and processing.

### RbdB is essential for SrbA activation.

**FIG 3:**
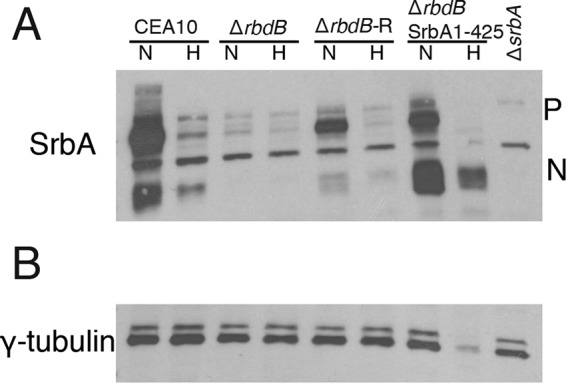
RbdB is critical for full processing of SrbA. Whole-cell extracts from the WT, Δ*rbdB*, Δ*rbdB*-R, SrbA1-425Δ*rbdB*, and Δ*srbA* strains grown under normoxic and hypoxic conditions were subjected to immunoblot analysis with antibodies against amino acids 1 to 275 of SrbA (A) and against γ-tubulin (B). P, precursor form of SrbA; N, cleaved and active form of SrbA.

In *A. fumigatus* and *S. pombe*, SrbA is synthesized as an ER-resident protein. To function as a transcriptional activator, SrbA is proteolytically cleaved and translocated to the nucleus, where it binds genes containing the SREBP DNA binding motif (12). Unlike mammalian SREBP pathways, in which site 1 and site 2 proteases cleave SREBPs in the Golgi lumen and in the first transmembrane domain, respectively (10), candidate proteases had not been characterized in *A. fumigatus* and *S. pombe*. Recently, Rbd2 in *S. pombe* was shown to be essential for Sre1 cleavage (13), and in *Aspergillus nidulans*, a novel aspartyl peptidase, SppA, was reported to cleave SrbA in its first transmembrane domain (19). To study the role of RbdB in the activation and cleavage of SrbA, we utilized an immunoblot approach using an antibody targeting the SrbA N terminus (amino acids 1 to 275) as previously described (12). As previously reported, the SrbA N terminus can be detected in normoxic shake flask cultures and also after a shift to hypoxic conditions in WT strains. However, our results indicate complete loss of the detectable SrbA N terminus in Δ*rbdB* under both normoxia and hypoxia conditions, suggesting that RbdB either directly cleaves SrbA or regulates SrbA N terminus protein levels (Fig. 3A). In support of the former hypothesis, expression of the first 425 amino acids of SrbA in Δ*rbdB* results in stable and abundant SrbA N terminus levels. Therefore, these data suggest that RbdB participates in the proteolytic processing of SrbA in *A. fumigatus.*

### The SrbA N terminus ameliorates the growth defect under hypoxic conditions, increased voriconazole susceptibility, and reduced extracellular siderophore production in Δ*rbdB*.

**FIG 4:**
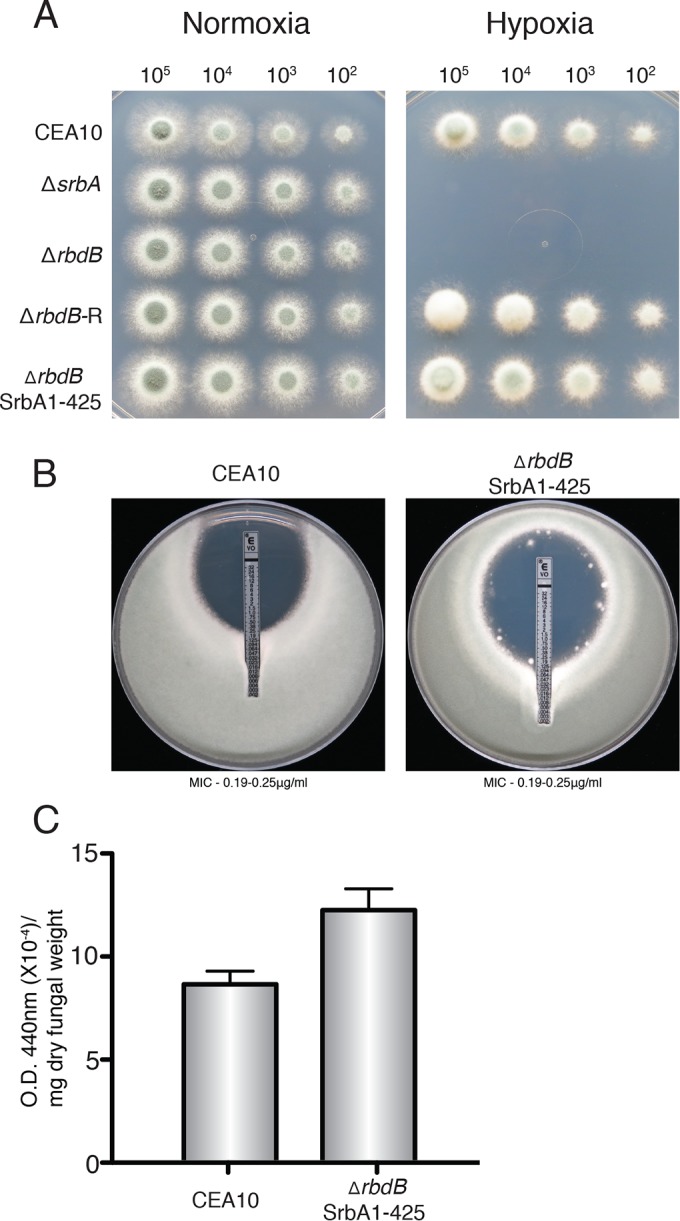
Expression of the bHLH domain of SrbA reverses the growth defect under hypoxic conditions, azole susceptibility, and iron homeostasis in Δ*rbdB*. (A) WT *A. fumigatus*, Δ*srbA*, Δ*rbdB*, Δ*rbdB*-R, and SrbA1-425Δ*rbdB* strains were serially inoculated into GMM. Growth was observed after 48 h of incubation at 37°C in complete darkness. The SrbA1-425Δ*rbdB* strain showed growth restoration compared to Δ*rbdB* under hypoxic conditions. (B) Voriconazole susceptibility of the WT and SrbA1-425Δ*rbdB* strains was measured with Etest strips. No difference in the MIC of voriconazole (0.19 to 0.25 µg/ml) for the strains tested was observed. (C) The SrbA1-425Δ*rbdB* strain showed complete restoration of extracellular siderophore production compared to Δ*rbdB*. Extracellular siderophore production was measured as described in Materials and Methods. O.D., optical density.

We have previously shown that the SrbA N terminus (amino acids 1 to 425) containing the bHLH DNA binding domain is functional, translocates to the nucleus, and largely restores SrbA functionality in Δ*srbA* (12). To further determine if the function of RbdB is associated with SrbA cleavage, we introduced SrbA1-425, the SrbA N terminus fragment, into Δ*rbdB* (SrbA1-425Δ*rbdB* strain). Consistent with our previous report, the SrbA1-425Δ*rbdB* strain grew under hypoxic conditions and no difference between its growth rate and that of the WT was observed (Fig. 4A). Furthermore, the susceptibility of the SrbA1-425Δ*rbdB* strain to voriconazole was comparable to that of the WT (MIC, 0.19 to 0.25 µg/ml) (Fig. 4B). In addition, with regard to extracellular siderophore production, approximately 83% more extracellular siderophore was produced by the SrbA1-425Δ*rbdB* strain than by Δ*rbdB*, which is similar to WT levels (Fig. 4C). Therefore, these data further support the hypothesis that RbdB is involved in proteolytic cleavage of SrbA to generate a functional SrbA transcription factor.

## DISCUSSION

Adaptation to hypoxia is a critical virulence attribute of pathogenic fungi, and the SREBP pathway plays an essential role in this adaptation in *A. fumigatus* and *C. neoformans* (1 – 3). Unlike mammals and *C. neoformans*, *A. fumigatus* lacks site 1 and site 2 proteases that cleave SREBPs to release the functional transcription factor (2, 19). In addition, *A. fumigatus* and associated members of the class *Eurotiomycetes* surprisingly apparently lack the critical SCAP. Therefore, the molecular mechanisms of *A. fumigatus* SREBP regulation remain enigmatic. As mutants lacking functional SREBP activity fail to cause disease and are more susceptible to antifungal drugs, a better understanding of SREBP cleavage and activation mechanisms in *A. fumigatus* is needed to target this pathway for therapeutic development (1). Here, we report that a rhomboid protease ortholog, RbdB, is essential for the function of *A. fumigatus* SREBP SrbA.

Mutants lacking *rbdB* failed to germinate under hypoxic conditions and were hypersusceptible to the triazole voriconazole, phenocopying Δ*srbA* (Fig. 1B and C). SrbA-dependent hypoxia-induced transcripts involved in ergosterol biosynthesis and iron acquisition were significantly reduced in Δ*rbdB* (Fig. 2B to D), suggesting that RbdB acts upstream of SrbA. Immunoblot analyses of SrbA levels in response to culture under normoxic and hypoxic conditions revealed that RbdB is critical for production of the transcription factor portion of SrbA (Fig. 3A). However, it is not known if RbdB physically interacts with SrbA, and the mechanism by which RbdB regulates production of the SrbA transcription factor awaits further investigation. Intriguingly, introduction of the SrbA transcription factor (amino acids 1 to 425) driven by the native *srbA* promoter into Δ*rbdB* increased levels of the SrbA N terminus compared to those in the WT. This result may suggest that posttranslational modifications of SrbA that regulate its stability occur prior to RbdB function. Ubiquitination by the DSc complex and phosphorylation by an unknown kinase(s) are potential mechanisms for future investigation.

The rhomboid class of serine proteases has been identified in all kingdoms of life (20). As homologs of site 2 proteases are absent from *A. fumigatus*, the rhomboid class of proteases seems to have an evolutionarily conserved function of RIP in fungi. In support of this hypothesis, the rhomboid protease 2 homolog *rbd2* was shown to be essential for Sre1 cleavage in *S. pombe* (13). Recently, SppA, an aspartyl protease, was identified as essential for SrbA cleavage in *A. nidulans* and *A. fumigatus*. SppA was shown to physically interact with SrbA, and SppA cleavage is succeeded by the activity of the DscE3 ligase complex (21). The exact role of the DSc E3 complex in *Aspergillus* SREBP processing is still unclear. However, the DSc complex, RbdB, and SppA are all essential for SrbA activation in *A. nidulans* and *A. fumigatus*. It is interesting that both Spp and rhomboid proteases cleave their substrates within transmembrane domains (22). With regard to RbdB, it is known that other substrates are cleaved by rhomboid proteases such as the Spitz ligand in *Drosophila melanogaster* (23), and thus we cannot rule out the possibility that RbdB is important in the regulation of other important factors that contribute to *A. fumigatus* virulence. Future experiments examining the SrbA1-425Δ*rbdB* strain *in vivo* may answer this question. What is clear is that the molecular mechanisms of SrbA regulation in filamentous fungi remain enigmatic and ill-defined.

In conclusion, we report a predicted rhomboid protease homolog, RbdB, as critical for activation of the *A. fumigatus* SREBP SrbA. As site 2 proteases have not been identified in *A. fumigatus*, identification of *rbdB* provides opportunities to better understand the molecular mechanisms regulating SREBP function in *A. fumigatus*. Future research will be focused on studying the mechanisms of SrbA activation through the interplay among RbdB, SppA, and the DSc complex. Given the complete loss of virulence and the marked increase in triazole drug susceptibility associated with mutations in these SREBP-associated genes, an increased understanding of the underlying molecular mechanisms is expected to yield novel therapeutic opportunities against this deadly fungal pathogen.

## MATERIALS AND METHODS

### Fungal strains, media, and growth conditions.

*A. fumigatus* CEA10 was used as the WT strain, and the *rbdB* (*AFUB_078750*) null mutant was generated in the CEA17 (*pyrG1*) background. Fungal stocks were stored as 30% glycerol stocks at −70°C. *A. fumigatus* was grown on GMM (14), and *N. crassa* was grown in Vogel’s minimal medium as previously described (24). Strains were cultured under normoxic conditions (21% O_2_, 5% CO_2_) or hypoxia (1% O_2_, 5% CO_2_) as previously described, unless specified otherwise (12).

### *N. crassa* deletion library screen.

The *N. crassa* complete genome deletion library was purchased from the Fungal Genetics Stock Center (25). Individual mutants were grown on Vogel’s minimal medium under normoxic and hypoxic conditions (0.2% O_2_, 5% CO_2_), and growth was monitored for 40 h. Mutants that failed to grow under hypoxic conditions were selected for further investigation.

### *A. fumigatus* strain construction.

The amino acid sequence of *N. crassa* Rbd2 (NCU02371.7) was used as the query sequence to search the *A. fumigatus* A1163 genome database available at AspGD with BLASTp. A single ortholog of Rbd2 was identified in *A. fumigatus* and named RbdB. A gene replacement construct for genetic replacement of the wild-type *rbdB* locus was generated by fusion PCR as described previously (26). Briefly, 1.2 kb of the 5′ and 3′ untranslated region (UTR) fragments of the *rbdB* gene were amplified with primers RAC3209 (5′-CGTCCGTGAGGGGCAAGAGTG-3′) and RAC3210 (5′-AGAGCATTGTTTGAGGCGACCGGTCCTAACTCACAGAATGCGCGACG-3′) and primers RAC3211 (5′-CGCATCAGTGCCTCCTCTCAGACCCTACCTCGCTCAGCAGGGAG-3′) and RAC3212 (5′-GTAGGGCACCGCTCTTCAGTGGC-3′), respectively. *Aspergillus parasiticus pyrG* was amplified from plasmid pSD38.1 with primers RAC2055 (5′-ACCGGTCGCCTCAAACAATGCTCT-3′) and RAC2056 (5′-GTCTGAGAGGAGGCACTGATGCG-3′) (26). The three resulting fragments were fused together with primers RAC3213 (5′-CGGAGCTTGCTGTGCCTCTGGC-3′) and RAC3214 (5′-GGCTTGAAAGACCCCTCCTCCACC-3′). Complete gene replacement was achieved by polyethylene glycol-mediated protoplast transformation with CEA17 as the host strain as previously described (27). Transformants were screened by PCR (data not shown) and Southern blot analyses as previously described (1).

### Generation of a reconstituted strain.

To generate a strain with reconstituted *rbdB*, the *rbdB* gene was amplified along with ~550 bp of the 5′ UTR and ~250 bp of the 3′ UTR with primers RAC3251 (5′-CTGGAAGAGCCCACTCAGGCAAAG-3′) and RAC3252 (5′-GTGAAAGGGCTTGGAGGGCAGG-3′). The resulting PCR product was transformed, as described above, into the Δ*rbdB* strain, and transformants were selected for the ability to grow under hypoxic conditions. The transformants that were able to grow under hypoxic conditions were confirmed by PCR and Southern blot analysis as previously described (1). The reconstituted strain was designated the Δ*rbdB*-R strain and represents an ectopic insertion of the reconstitution construct into the Δ*rbdB* strain.

### Generation of the SrbA1-425Δ*rbdB* strain.

The *srbA* genomic sequence coding the first 425 amino acids (1,275 nucleotides), followed by a stop codon along with 1.2 kb of the 5′ UTR, was amplified from genomic DNA with primers RAC2051 (5′-ACGTTCGGTAGCGCCAATGGCAAG-3′) and RAC3282 (5′-TCAAATTGCGCGCGCGTGCCAAGACG-3′). The resulting DNA fragment was transformed into the Δ*rbdB* strain, and transformants were selected for growth under hypoxic conditions. Transformants growing under hypoxic conditions were confirmed by PCR and Southern blot analysis as described previously (1). The resulting strain was designated the SrbA1-425Δ*rbdB* strain*.*

### Morphological analysis

For morphological analysis, WT *A. fumigatus* (CEA10), Δ*rbdB*, Δ*rbdB*-R, and SrbA1-425Δ*rbdB* strains were serially inoculated into GMM. Images were captured after 48 h of growth in complete darkness under the respective conditions.

### Antifungal susceptibility testing.

Antifungal susceptibility testing was done as described previously (28). Briefly, 5 ml of molten agar containing 10^5^ spores of the WT *A. fumigatus*, Δ*rbdB*, Δ*rbdB*-R, or SrbA1-425Δ*rbdB* strain was overlaid on GMM plates. Voriconazole Etest strips (BioMérieux) were placed in the centers of the plates, and the plates were incubated for 48 h in the dark. MICs based on the zones of clearance were recorded. The experiment was performed with biological triplicates.

### Extracellular siderophore assay.

Extracellular siderophore production was measured by a modified form of the assay previously described (29). Briefly, fungal strains were grown in 50 ml of liquid GMM without iron under normoxic conditions at 37°C with constant shaking at 200 rpm in complete darkness; this was followed by a shift to hypoxia under the same conditions for an additional 24 h. Fungal biomass was collected via vacuum filtration and lyophilized. Culture supernatants were collected and adjusted to pH 6.5 with NaOH and/or HCl and filtered through low-protein-binding filters (0.45-µm pore size; Millipore). Five microliters of FeSO_4_ dissolved in 5mM HCl was added to 95 µl of culture filtrates to a final concentration of 1.5 mM iron in 96-well plates, and the plates were incubated for 30 min at room temperature. Optical density at 440 nm was measured and normalized to control wells incubated with HCl alone. Siderophore production per milligram of fungal dry weight was measured.

### Immunoblotting.

Cultures containing 50 ml of GMM were inoculated with the WT CEA10, Δ*rbdB*, Δ*rbdB*-R, or SrbA1-425Δ*rbdB* strain, respectively, and incubated for 16 h under normoxic conditions; this was followed by a shift to hypoxia for 2 h. Mycelia were harvested by vacuum filtration and washed with a saline solution containing 1 mM phenylmethylsulfonyl fluoride and 1% dimethyl sulfoxide, flash frozen with liquid N_2_, and lyophilized. Protein extraction, SDS-PAGE, transfer, and detection were done as previously described (1).

### RNA extraction and quantitative RT-PCR.

*A. fumigatus* strains were grown under the same conditions as for immunoblot assays. RNA extraction, quantitative RT-PCR, and data analyses were done as previously described (18). The data from three independent biological replicates were analyzed, and statistical significance was assessed by one-way analysis of variance (ANOVA).

### Murine virulence assay.

Murine virulence tests were done as described earlier, with some modifications (12). Briefly, female 7-week-old CD-1 mice weighing 28 to 30 g were immunosuppressed with a single 40-mg/kg subcutaneous injection of triamcinolone acetonide (Kenalog; Bristol-Myers Squibb) 1 day prior to inoculation with *A. fumigatus* conidia. Mice were inoculated intranasally with 2 × 10^6^ spores of the WT CEA10, Δ*rbdB*, or Δ*rbdB*-R strain, respectively, in phosphate-buffered saline (PBS). Mice were monitored three times daily, and their survival was monitored for 14 days postinoculation. Statistical comparison of the associated Kaplan-Meier curves was conducted with log rank tests as previously described (12).

### Ethics statement.

The Guide for the Care and Use of Laboratory Animals of the National Research Council was strictly followed for all animal experiments. The animal experiment protocol was approved by Institutional Animal Care and Use Committee at Dartmouth College (protocol cram.ra.1).
