# Erratum for Dhingra et al., “RbdB, a Rhomboid Protease Critical for SREBP Activation and Virulence in Aspergillus fumigatus”

**DOI:** 10.1128/mSphere.00678-19

**Published:** 2019-09-25

**Authors:** Sourabh Dhingra, Caitlin H. Kowalski, Arsa Thammahong, Sarah R. Beattie, Katherine M. Bultman, Robert A. Cramer

**Affiliations:** a Department of Microbiology and Immunology, Geisel School of Medicine at Dartmouth, Hanover, New Hampshire, USA

## ERRATUM

Volume 1, no. 2, e00035-16, 2016, https://doi.org/10.1128/mSphere.00035-16. This article was published on 2 March 2016 with the second author’s surname misspelled. The byline has been updated in the current version, posted on 20 September 2019.

